# The role played by granulocyte colony stimulating factor (G-CSF) on women submitted to *in vitro* fertilization associated with thin endometrium: systematic review

**DOI:** 10.5935/1518-0557.20200025

**Published:** 2020

**Authors:** Mylena Naves de Castro Rocha, Rodopiano de Souza Florêncio, Rosane Ribeiro Figueiredo Alves

**Affiliations:** 1 Humana Medicina Reprodutiva - Goiânia, GO, Brazil

**Keywords:** granulocyte colony-stimulating factor, *in vitro* fertilization, endometrium

## Abstract

**Objective:**

To provide evidence available in the literature on the role of granulocyte colony stimulating factor (G-CSF) in women submitted to in vitro fertilization, with repeated implantation failure associated with thin endometrium.

**Methods:**

Systematic review of the use of G-CSF, as part of assisted reproduction techniques in women with repeated embryo implantation failures associated with thin endometrium. The study was carried out in the PubMed, BIREME and Elsevier databases from 2008 to 2018, in English, Spanish and Portuguese.

**Results:**

We included all the studies, which used intrauterine G-CSF. We found an increase in endometrial thickness in eight of the 10 studies included. Of these, the implantation rate improved significantly in two studies, but the gestation rate increased in only one. We found the highest rates of implantation (32%) and pregnancy (48%) in a non-randomized clinical trial. On the other hand, two other studies did not demonstrate an increase in endometrial thickness and in pregnancy rates in patients with thin endometrium submitted to the assisted reproduction in frozen embryo transfer cycles.

**Conclusion:**

Studies published so far point to a positive influence on the use of G-CSF in relation to the improvement in endometrial receptivity and pregnancy rates. Therefore, there is a need for further studies to determine whether to use it, as well as the period, route of administration, dosage and duration of treatment.

## INTRODUCTION

Despite advances in assisted reproduction, the rates of a well-succeeded embryo implantation are still low. Embryo quality and endometrial receptivity, apart from a suitable embryo transfer technique, may influence the success of such implantation (Zenclussen & Hämmerling, 2015; Kushnir *et al.*, 2017). However, the morphological quality of an embryo is not a guarantee of a well-succeeded implantation. Therefore, the exclusion of an embryo with a better chance of implantation may occur, just because it was not considered the one with the best morphological aspect at the moment of evaluation (Donadio *et al.*, 2012).

Conversely, it is believed that the adequate endometrial thickness could make the endometrial cells change into decidual easier, as well as the invasion of blastocysts and a timely placenta growth (Zenclussen & Hämmerling, 2015). However, there is no agreement in the literature regarding endometrial thickness to characterize a receptive endometrium. A thin endometrium is seen more often in women aged over 40, probably due to vascularity decrease. A 2.4% to 5% prevalence of thin endometrium has been reported in women under 40 years of age, and 25% in women over 40 in natural cycles (Sher *et al.*, 1991; Kasius *et al*., 2014).

There are studies which indicate a thickness threshold below 7mm (Mahajan & Sharma, 2016; Cavalcante *et al.*, 2015), yet others report 6mm (Shapiro *et al.*, 1993; Kunicki *et al.*, 2014) or 8mm (Gingold *et al.*, 2015). On the other hand, a study reports clinical pregnancy with 4mm of endometrial thickness (Check *et al.*, 2016), which brings about the possibility that the endometrial receptivity may not necessarily be related to the endometrial thickness. In spite of not having a consensus, endometrial thickness has been used to predict the likelihood of pregnancy in assisted reproduction cycles.

Today, there are pieces of evidence that the embryonic implantation process turned easier by immune cells, growth factors, cytosines, and hormonal changes (Kunicki *et al.*, 2014; Davari-Tanha *et al.*, 2016; Eftekhar *et al.*, 2016a). G-CSF is a hemanopoietic cytosine produced in the reproductive system, at the maternofetal interface, during embryo implanting, which stimulates granulocyte proliferation and differentiation. It has been suggested that this cytosine could, therefore, play a role both on the decidua and the placental function (Salmassi *et al.*, 2004; Li *et al.*, 2014; Cavalcante *et al.*, 2015). G-CSF for clinical use is mainly indicated to reduce neutropenia duration and fevered neutropenia incidence in patients with non-myelogenic neoplasia, undergoing cytotoxic chemotherapy. Besides this, it is also indicated to reduce neutropenia duration and after-effects in patients submitted to myeloablative therapy followed by bone marrow transplant (Wurfel, 2015). Synthetic G-CSF differs from its natural counterpart for presenting an additional N-methionine terminal residue and for the lack of O-Glycogenesis. In Brazil this drug is traded under the name Filgastrim (Granulokine; Roche), presented in pre-bottled syringes holding 0.5mL injectable solution, containing 300µg, which comprises 30 million units.

The first evidence of improvement on *in vitro* fertilization embryo implanting rates and higher G-CSF concentration in follicular liquid was reported in 2005 (Salmassi *et al.*, 2005). Since that time, some studies have evaluated G-CSF usage in a systemic form via subcutaneous injection or directly in the endometrium via intrauterine injection (Kunick *et al.,* 2014, Li *et al*., 2014), in women with recurrent spontaneous abortion and repeated implantation failures. Others show pregnancy improved results (Santjohanser *et al.*, 2013; Eftekhar *et al.*, 2016b), even in those with thin endometrium (Gleicher *et al.*, 2013; Lucena & Moreno-Ortiz, 2013). Thus, the objective of this study was to systematize the literature evidence on the use of G-CSF and pregnancy rates in women submitted to assisted reproduction techniques (ART) with repeated failures associated with thin endometrium.

## MATERIALS AND METHODS

This systematic review study included papers published in English, Spanish, and Portuguese, which investigated the use of intrauterine or subcutaneous G-CSF in cases of implantation failure, associated with thin endometrium in the context of human assisted reproduction. We searched in PubMed, Bireme and Elsevier databases, using the following keywords: "granulocyte colony-stimulating factor” [MeSH Terms] AND "endometrium"[MeSH Terms] AND ("humans"[MeSH Terms] AND (English [lang] OR Portuguese[lang] OR Spanish[lang])) in a-10 year period (from January 2008 to March 2018).

Two independent authors read the titles and abstracts in order to check for duplicates and to meet the pre-established inclusion criteria. Afterwards, they read the potentially eligible papers entirely. Those papers, which despite reporting the use of G-CSF in human reproduction did not evaluate its impact on endometrial thickness and on pregnancy rates, we discarded. The data was extracted from the text, tables, and graphs in the studies included. We collected information such as study type, place and year of publication, number and age of participants, the timeframe and G-CSF administration via, endometrial thickness before and after G-CSF, and pregnancy rates.

## RESULTS

**Figure 1:**
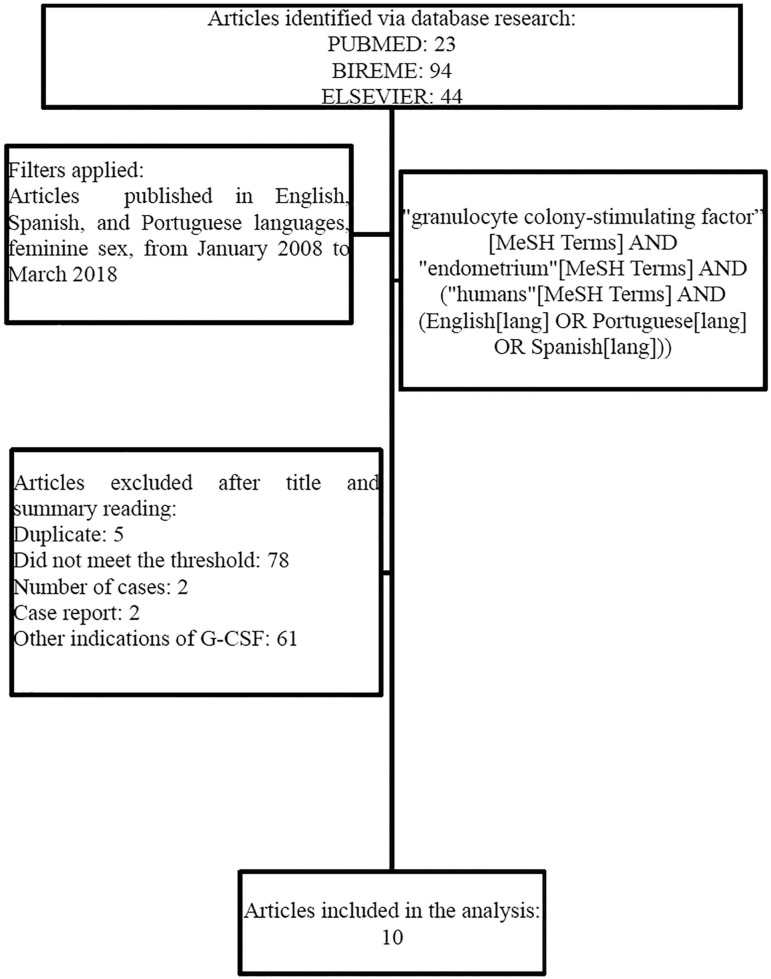
Flowchart of the papers included in the systematic review study

We selected 161 papers: 23 from PubMed, 94 from Bireme, and 44 from Elsevier (Figure 1). Five papers were taken off for being duplicated, 78 for not addressing the G-CSF regarding repeated failures associated with thin endometrium, and 68 for addressing other indications regarding the use of G-CSF. In our study, 10 papers were included, namely, two randomized clinical trials (Eftekhar *et al*., 2016b; Sarvi *et al.,* 2017), three not-randomized clinical trials (Xu *et al*., 2015; Tehraninejad *et al.,* 2015; Eftekhar *et al.*, 2014), three prospective cohort studies (Shah *et al.,* 2014; Gleicher *et al.,* 2011; 2013) and two cross-sectional studies (Mishra *et al*., 2016; Kunicki *et al.,* 2014).

Out of these 10 studies, seven were published in the Asian continent, one in the European, and two in the American continent (Chart 1). These papers were published from 2011 to 2017 and included 475 participants with thin endometrium and repeated failures in the assisted reproduction techniques to whom, G-CSF was employed. The average age range of the participants included in the published studies was from 30.5 to 40.5. All of the 10 studies included utilized G-CSF via uterine at a 300mcg dosage. The G-CSF application day concerning the menstrual cycle period varied among the studies (Chart 1).

**Chart 1:** List of the studies that evaluated the use of G-CSF in women submitted to assisted fertilization, which held thin endometrium and repeated failures

First author/Year Geographic Region	Study Type	Participants number	Average age	G-CSFmethod of use Date	Endometrium thikness after G-CSF (average)	Pregnancy rates (%)
Sarvi *et al*., 2017 Iran	RCT	TG: 13 CG: 15	TG:31.2 CG: 31.6	TG: 300 mcg IU GC: Saline	TG: 5±1.4 mm	CG: 20 TG: 15.3 **
Eftekhar *et al*., 2016b Iran	RCT	TG: 44 CG: 45	TG: 32.5 CG: 31.7	300 mcg IU	CG: 8.8 TG: 9.1 **	TG: 28.8 CG: 1.3 *
Xu B *et al*., 2015 China	Non-randomized CT	TG: 41 CG: 65	TG: 31.4 CG: 32.0	300 mcg IU	5.7mm antes 8.4 mm após*	TG: 48 CG: 25*
Tehraninejad *et al*., 2015 Iran	Non-randomizedCT	15	35.13	300 mcg IU eggs collecting day	3.6mm antes 7.1mm após*	20
Eftekhar *et al*., 2014 Iran	Non-randomized CT	TG: 34 CG: 34	TG: 30.8 CG: 28.6	300 mcg IU 12th to 13th day	5.6 CG 5.8: TG **	CG:28.6 TG:30.8 **
Shah *et al*., 2014 India	Prospective cohort study	EG: 231 NEG: 117	33.5	300 mcg IU 10 days from estrogen onset	< 8mm before 10.9mm after *	37
Gleicher *et al*., 2013 USA	Prospective cohort study	21	40.5	300 mcg IU on hCG day	5.7mm before 9.3mm after*	19.1
Gleicher *et al*., 2011 USA	Prospective cohort study	4	38.3	300mcg IU 48 h beforeET	4.9mm before 8.7mm after*	100 (1 ectopic)
Mishra *et al*., 2016 India	Cross-sectional study	35	30.5	300 mcg IU on 14th day of cycle	5.9mm before 6.6mm after *	Zero
Kunicki *et al*., 2014 Poland	Cross-sectional study	37	34.7	300 mcg IU on hCG day	6.7 before 8.4 after *	19.1

RCT: Randomized clinical trial. IU: intrauterine; ET: Embryo Transfer; CT: Clinical Trial; SD: Standard Deviation; IR: implantation Rate;

* Significant;

** not significant.

TG: Treated Group. CG: Control Group, EG: Exposed Group; NEG: Non-Exposed Group.

Three out of the five clinical trials evidenced significant increases on the endometrial thickness with the use of G-CSF (Sarvi *et al.,* 2017; Xu *et al*., 2015; Tehraninejad *et al*., 2015); three prospective cohorts (Shah *et al*., 2014; Gleicher *et al*., 2011; 2013) and two out of the cross-sectional studies (Mishra *et al*., 2016; Kunicki *et al*., 2014) included in the present review. The pregnancy rates in the studies, which showed endometrial thickness significant increase, ranged from 19.1% to 37.0% (Chart 1).

Three non-randomized clinical trials (Eftekhar *et al.*, 2014; Tehraninejad *et al.*, 2015; Xu *et al.*, 2015) and two randomized clinical studies (Eftekhar *et al.*, 2016b; Sarvi *et al.*, 2017) showed implantation rate increase. Despite the small number of participants involved in the included randomized trials, the implantation rate ranged from 10 to 17% and the pregnancy was 29% after G-CSF treatment. Three out of the ten included studies did not evidence improvements in pregnancy rates (Eftekhar *et al*., 2014; Mishra *et al.*, 2016; Sarvi *et al.*, 2017). As for the other seven studies, the rates ranged from 19 to 37% (Gleicher *et al.*, 2011; 2013; Kunicki *et al.*, 2014; Shah *et al*., 2014; Tehraninejad *et al.*, 2015; Xu *et al.*, 2015; Eftekhar *et al*., 2016b). The highest clinical pregnancy rate (28.8%) was found in the randomized clinical trial (Eftekhar *et al.*, 2016b) and, the rate in the implantation group, treated with G-CSF, was 17%; whereas in the control group, it was 5% (*p*<0.05).

## DISCUSSION

Intrauterine G-CSF infusion in assisted reproduction cycles aims at increasing the endometrial receptivity and, thus, reshape and increase endometrium thickness. Therefore, it would help the embryo transfer and clinical pregnancy rates (Eftekhar *et al.*, 2014; Kunicki *et al.*, 2014; Eftekhar *et al.*, 2016a; b). Nonetheless, literature data is not conclusive regarding what it considers as thin or unresponsive endometrium. Some studies state that pregnancy takes place when the endometrium reaches over 7mm (Gleicher *et al.*, 2013; Gingold *et al*., 2015), and others say that more than 9mm is required (Kasius *et al.*, 2014; Mahajan & Sharma, 2016). Besides this, there is strong evidence that the thin endometrium is not necessarily a factor, which hinders, a well-succeeded embryo implanting. There is evidence that the thin endometrium is not necessarily a factor preventing successful embryo implantation, although it may negatively affect pregnancy after embryo transfers (Gingold *et al.*, 2015; Mahajan & Sharma, 2016).

The studies included in this review, demonstrated an effect considered moderate, on the unresponsive endometrium treatment, with low implantation and pregnancy rates. G-CSF use as an additive in assisted reproduction treatment, aiming at enhancing endometrial receptivity, is new. It is expected, though, that G-CSF will be an outstanding agent in assisted reproduction (Eftekhar *et al.*, 2016a; Sarvi *et al.*, 2017).

Randomized clinical trials are taken as standard of excellence to evaluate the efficacy of an intervention. So, the results presented by Eftekhar *et al*. (2016b) and Sarvi *et al*. (2017), concerning thin endometria and low implantation and pregnancy rates, provide resources to carry out trials with higher number of participants aiming at evaluating the G-CSF efficacy. Another important factor to be noticed is that there was no homogeneity on the treatment utilized in the evaluated trials, neither with regards to intrauterine infusion day nor number and dose applied. Therefore, there is a need for better evidence about number and dose, and on the cycle phase they should be administered. In addition to this, the timeframe between intrauterine infusion and endometrium evaluation is not well-grounded in literature. Gleicher *et al*. (2011), did the re-evaluation at 48h, time much shorter than the one reported by Kunicki *et al*. (2014).

One of the earlier publications regarding its use dates back to 2011 (Gleicher *et al.*, 2011). However, over these seven years, few clinical essays were performed with few participants, which constraints the conclusion with regards to the benefit or not of its use, in assisted reproduction. Thus, despite improvements in endometrial thickness associated with increases in pregnancy rates, confirmed in eight studies in the present review (Gleicher *et al.*, 2011; 2013; Shah *et al.*, 2014; Kunicki *et al.*, 2014; Tehraninejad *et al.*, 2015; Xu *et al.*, 2015; Mishra *et al.*, 2016; Sarvi *et al.*, 2017), the results are not sufficient yet to provide a robust evidence for the use of G-CSF in patients with thin endometria, as well as repeated failures both at implantation and pregnancy rates. In a cohort study, all of the treated patients improved their endometrial thickness at an average of 3.54 mm upon treatment and, at the moment of the publishing they had an ongoing pregnancy (Gleicher *et al*., 2011).

Thus, the best moment for this evaluation still remains as a question to be answered. Likewise, there is a need for long-term studies to evaluate the impact of the treatment on the future health of the babies born. Amongst the results limitation, we point out the small number of studies, all of them with small sampling, and the variability of evaluated criteria, as well as low levels of evidence, based on the study design of the included papers. Thus, in order to indicate the clinical use of the G-CSF in patients with implantation failure due to thin endometrium, there is a need for further studies.

## CONCLUSION

The pieces of evidence in the literature suggest a positive influence of G-CSF on improving endometrial receptivity and pregnancy rates. Notwithstanding, the literature evidence is conflicting and of hard comparison because of the small number of studies addressing the theme, as well as for the different types of studies. There is a must for more controlled randomized studies involving a larger number of participants to make it possible to establish the correct prescription, as well as the suitable dose and the treatment timeframe.
